# Looking At the Past and Heading to the Future: Meeting Summary of the 6^th^ European Workshop on Plant Chromatin 2019 in Cologne, Germany

**DOI:** 10.3389/fpls.2019.01795

**Published:** 2020-02-07

**Authors:** Jordi Moreno-Romero, Aline V. Probst, Inês Trindade, Kalyanikrishna, Julia Engelhorn, Sara Farrona

**Affiliations:** ^1^Centre for Research in Agricultural Genomics (CRAG), CSIC-IRTA-UAB-UB, Campus UAB, Bellaterra, Barcelona, Spain; ^2^GReD, Université Clermont Auvergne, CNRS, INSERM, BP 38, Clermont–Ferrand, France; ^3^Institute for Biochemistry and Biology, University of Potsdam, Potsdam, Germany; ^4^Institute for Biology, Freie Universität Berlin, Berlin, Germany; ^5^Institute for Molecular Physiology, Heinrich-Heine-Unverisität, Düsseldorf, Germany; ^6^Max Planck Institute for Plant Breeding Research, Cologne, Germany; ^7^Plant and AgriBiosciences Centre, Ryan Institute, NUI Galway, Ireland

**Keywords:** EWPC2019, chromatin, epigenetics, transcription, nucleus

## Abstract

In June 2019, more than a hundred plant researchers met in Cologne, Germany, for the 6^th^ European Workshop on Plant Chromatin (EWPC). This conference brought together a highly dynamic community of researchers with the common aim to understand how chromatin organization controls gene expression, development, and plant responses to the environment. New evidence showing how epigenetic states are set, perpetuated, and inherited were presented, and novel data related to the three-dimensional organization of chromatin within the nucleus were discussed. At the level of the nucleosome, its composition by different histone variants and their specialized histone deposition complexes were addressed as well as the mechanisms involved in histone post-translational modifications and their role in gene expression. The keynote lecture on plant DNA methylation by Julie Law (SALK Institute) and the tribute session to Lars Hennig, honoring the memory of one of the founders of the EWPC who contributed to promote the plant chromatin and epigenetic field in Europe, added a very special note to this gathering. In this perspective article we summarize some of the most outstanding data and advances on plant chromatin research presented at this workshop.

## Introduction

Last year, the Max Planck Institute for Plant Breeding Research in Cologne hosted the 6^th^ European Workshop on Plant Chromatin (EWPC). A total of 110 researchers met to present the most recent focuses, advances, and challenges in the plant chromatin and epigenetics field during this 2-day workshop that comprised more than 25 standard talks and a similar number of short PechaKucha-style talks. Many other topics were talked over during the poster sessions in which the participants had the opportunity to discuss new discoveries and concepts in plant chromatin science in a thriving atmosphere.

**Figure 1:**
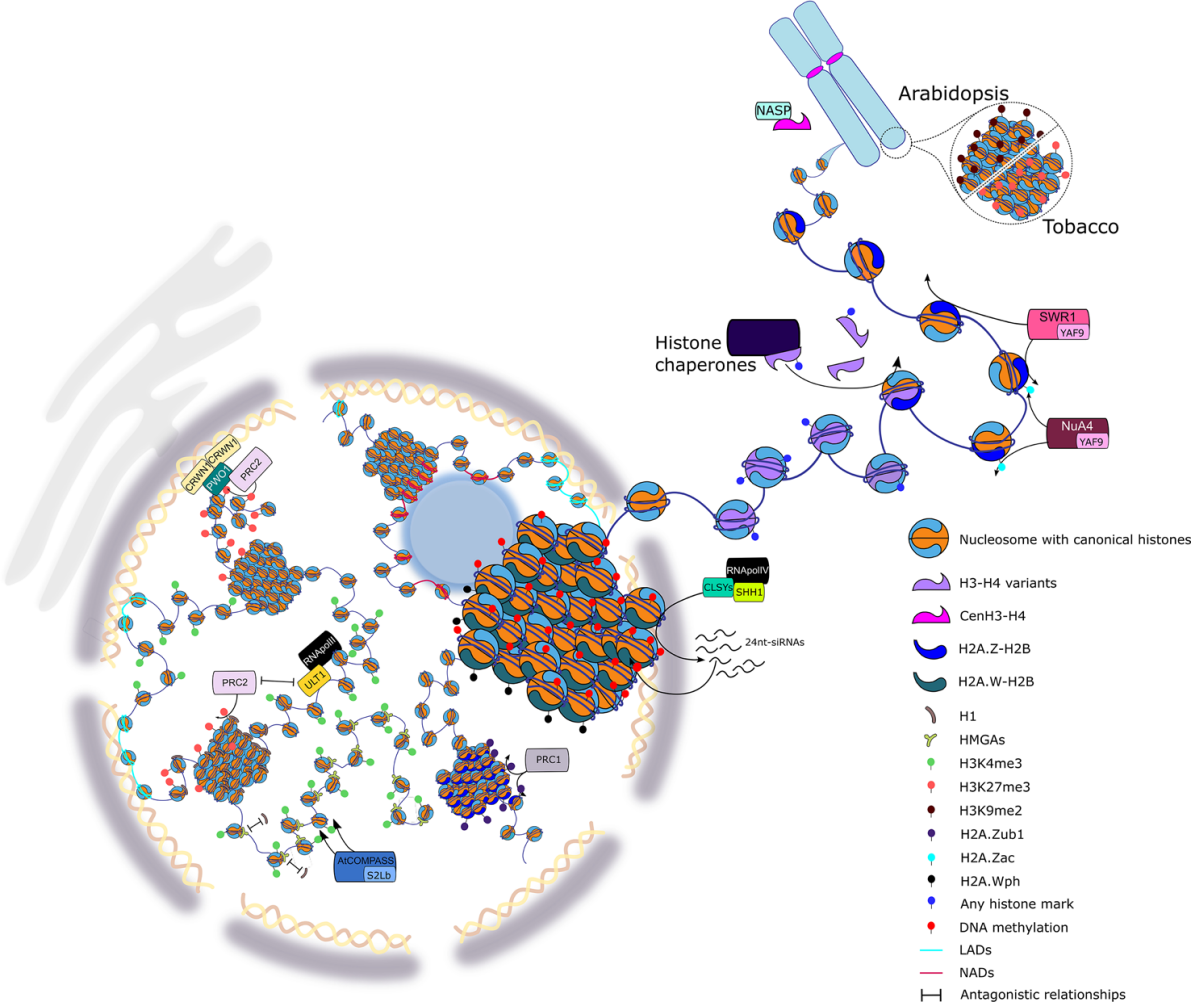
Highlights of the European Plant Chromatin Workshop 2019. Chromatin organization is a central player in controlling gene expression and concomitantly regulating plant development and plant responses to the environment. The scheme illustrates some of the aspects of plant chromatin organization presented at the EWPC ranging from local chromatin changes touching the bricks of the nucleosome to higher-order chromatin organization. At the level of the nucleosome, modifications of the DNA molecule and histone proteins were discussed, such as the regulation of DNA methylation involving the CLASSY (CLSY) proteins, the incorporation of specific variants of histones H1, H3, and H2A through dedicated histone chaperone complexes, and the dynamics of non-histone DNA-binding proteins, such as HIGH MOBILITY GROUP A (HMGA). Post-translational modifications of histones are set by specific complexes exemplified here by COMPLEX PROTEINS ASSOCIATED WITH SET1 (COMPASS), involved in H3K4me3, Nucleosome Acetyltransferase of Histone H4 (NuA4), in H2A.Z acetylation, and Polycomb Repressive Complex 1 (PRC1), in H2A.Z monoubiquitination. The role of chromatin remodelers in the deposition of histones is also depicted through H2A.Z-mediated deposition by the SWI/SNF-Related protein 1 (SWR1) complex. Modification of H2A.W by phosphorylation and its association to the heterochromatin was observed. The interplay between repressive modifications set by PRC2 and the antagonizing activity of ULTRAPETALA1 (ULT1) allows for a dynamic transcriptional regulation. Finally, the formation of specific chromatin domains in the nucleus, such as telomeres, nucleolus/lamina associated domains (NADs/LADs), or the association of chromatin domains *via* PWWP INTERACTOR OF POLYCOMB (PWO1) to CRWN1, a plant lamina component, were presented.

Several talks emphasized the complexity of chromatin organization within the three-dimensional space of the nucleus and presented cutting-edge techniques developed to provide a deeper and higher-resolution view of chromatin structure (**Figure 1**). As in previous EWPCs, histone variants and histone marks were an important theme for many research laboratories. Considerable progress has been made in recent years to understand their links to transcriptional regulation. Also, of note have been the advances in our understanding of the proteins and complexes that are involved in the deposition of histone variants and marks, which, additionally, may act as readers of these chromatin features.

Current challenges that have arisen from issues such as food security and climate change have added a new dimension to the study of epigenetic regulation of plant traits and epigenetic inheritance of transcriptional stages. For that reason, the link between chromatin dynamics, gene expression, and plant developmental adaptation to the environment was also substantially addressed in the meeting. To advance in this field, analyses of chromatin architecture changes at different developmental stages and the tissue- or cell-specific level that have been technically challenging were presented.

Julie Law from the Salk Institute (La Jolla, USA) was invited to present the keynote lecture, which highlighted some of the most important past and present contributions to the DNA methylation field from her laboratory. Julie gave an overview of the crucial roles played by DNA methylation in gene regulation and transposon silencing. In addition, she reported that a family of four putative chromatin remodeling factors, CLASSY (CLSY) 1–4, associate with the RNA-directed DNA methylation (RdDM) pathway components Pol-IV and SAWADEE HOMEODOMAIN HOMOLOG 1 (SHH1) (Law et al., 2011). Further recent studies showed that CLSY proteins function individually as locus-specific regulators of RdDM and in global regulation of DNA methylation patterns in Arabidopsis (Zhou et al., 2018). The next phase of the Law’s laboratory work aims to identify the roles of CLSY proteins in controlling DNA methylation patterns in a tissue-specific manner.

The EWPC was also the perfect venue for honoring the memory of Professor Lars Hennig who has recently passed away (Mozgová et al., 2018a). Together with Claudia Köhler and Valérie Gaudin, he established the EWPC in 2009 as one of the main gathering platforms for the plant epigenetic research community in Europe, bringing his enthusiasm and passion on plant science to these workshop series. During this tribute session, Lars’ colleagues and alumni shared with the audience his impact and vision on the chromatin and epigenetics field.

This perspective article summarizes the main topics discussed during the EWPC 2019 and provides insight into the future paths that the plant epigenetic community will follow in the next years. We thank all the laboratories, which have contributed to the EWPC with recently published or unpublished data, and we apologize to the researchers whose work could not be cited due to space limitations.

## Session 1: a View on Chromatin, Techniques, and Nuclear Structure

The key bearer of genetic information in eukaryotic cells is chromatin, which is non-randomly distributed inside the nucleus and shows an extraordinary degree of compaction and spatial organization. Nuclear organization is achieved by many factors, including histone proteins, modifiers and readers, as well as structural components of the nuclear periphery and nuclear bodies, which together dynamically control the nuclear architecture and may form nuclear domains (Sexton and Cavalli, 2015). The first session of the EWPC meeting dealt with the role of these factors in chromatin and nuclear organization.

The core histones have been structurally conserved through evolution and have evolved to accomplish two conflicting and yet vital tasks: on one hand, the long DNA molecules have to be packaged within the limits of the eukaryotic nucleus, preventing knots and tangles and protecting the genome from physical damage; on the other hand, the information that is encoded in the DNA needs to be accessed at appropriate times (Rosa and Shaw, 2013). The linker DNA between nucleosomes is bound by linker histones H1 (Rutowicz et al., 2015; Kotliński et al., 2017) whose role is much less understood than core histones. A recent study presented by Célia Baroux (Zurich, Switzerland) provided a multi-scale functional analysis of Arabidopsis linker histones. The work, done in collaboration with the laboratories from Andrzej Jerzmanowski (Warsaw, Poland) and Fredy Barneche (Paris, France), showed that H1-deficient plants are viable but exhibit phenotypes in seed dormancy, flowering time, as well as lateral root and stomata formation. In addition to a role in heterochromatin compaction, H1 seems to regulate nucleosome distribution over gene bodies. Yet, the authors showed that H1-mediated chromatin organization may act downstream of transcriptional control for a large number of loci in Arabidopsis. In addition, a new connection was found between H1 and H3K27me3. The findings suggest that H1 may act as a chromatin organizer favoring the maintenance of this epigenetic mark as well as others (Rutowicz et al., 2019).

Frédéric Pontvianne (Perpignan, France) focused on the nucleolus, the largest nuclear body, which is well known as the site of ribosomal RNA (rRNA) gene transcription, rRNA processing, and ribosome biogenesis (Boisvert et al., 2007). In a previous study, Frédéric and co-workers identified chromatin regions associated with the nucleolus, termed Nucleolus Associated Domains (NADs). NADs are primarily genomic regions with heterochromatic signatures and include transposable elements (TEs), sub-telomeric regions, and mostly inactive protein-coding genes (Pontvianne et al., 2016). Recent data now suggest that the rRNA gene copy number impacts the organization of NADs, and this suggests a role of nucleolus organizer regions (NORs) in establishing domains of inactive chromatin associated with the nucleolus (Picart-Picolo et al., 2019).

Similar to the nucleolus, the nuclear periphery is another compartment within the nucleus that plays a crucial role in chromatin organization and nuclear architecture. Kalyanikrishna (Berlin, Germany) presented data showing a putative link between Polycomb Group (PcG)-mediated repression and the nuclear periphery in Arabidopsis. PWWP INTERACTOR OF POLYCOMB (PWO1) is a PWWP-domain containing protein able to interact with any of the three possible POLYCOMB REPRESSIVE COMPLEX 2 (PRC2) histone methyltransferases in Y2H, and PWO1-CURLY LEAF (CLF) interaction was confirmed in planta (Hohenstatt et al., 2018; Mikulski et al, 2019). Among the putative interactors of PWO1, CROWDED NUCLEI1 (CRWN1) has been identified (Mikulski et al, 2019). CRWN1 is a coiled coil analog of lamin proteins, whose absence alters nuclear morphology (Wang et al., 2013), and a set of H3K27me3 targets were upregulated in *crwn1 crwn2* double mutants in Arabidopsis. The interaction between PWO1 and CRWN1 suggests a role of the nuclear periphery in PRC2-mediated gene regulation in Arabidopsis (Mikulski et al., 2019). The Schubert laboratory continues to work on characterizing putative interactors involved in this pathway.

The post-translational modifications of telomere histones in plants have been investigated by Katerina Adamusova (Brno, Czech Republic). Among the canonical and non-canonical telomeres in plants, the authors found two kinds of epigenetic patterns regardless of the differences in telomere length and telomeric sequences used. One of them corresponds to the Arabidopsis-like pattern, where telomere histones are marked predominantly with H3K9me2. The other one is the tobacco-like pattern marked predominantly with H3K27me3 (Adamusová et al., 2019).

Hua Jiang (Gatersleben, Germany) discussed the role of AT-hook proteins in the regulation of gene expression by mediating the H3K9me2 heterochromatic mark at the nuclear matrix-associated regions (MARs). They identified AT-Hook Like 10 (AHL10), a member of the AT-hook family in Arabidopsis, and the SET domain containing SU(VAR)3-9 homolog (SUVH9) as interacting partners of ADMETOS (ADM), which functions in establishing the postzygotic hybridization barrier in Arabidopsis. Significantly increased expression of *ADM* and *AHL10* in Arabidopsis triploid seeds results in H3K9me2 hypermethylation in MARs. Furthermore, AHL10-mediated H3K9me2 hypermethylation at MARs is independent of DNA methylation (Jiang et al., 2017). Apart from AHL10, the authors found that the overexpression line of another *AHL* also has increased H3K9me2 levels at TEs in sporophytic tissues, indicating a similar role for other members of this family.

## Session 2: Chromatin, Inheritance, and Generation Changes

Recent advances in our understanding of inter-generational inheritance of epigenetic and chromatin marks have revealed a variety of plant peculiarities, rendering this topic an exciting field of study with impact on our fundamental understanding of inheritance, phenotypic plasticity, population dynamics, and evolution (Köhler and Springer, 2017; Miryeganeh and Saze, 2019). Nevertheless, many open questions remain concerning what epigenetic information is inherited, the mechanisms of inheritance, and the processes involved in eventual reprogramming to prevent inheritance. To shed light on these questions, an enhanced understanding of gene regulation in gametophytes is vital. Sara Simonini (Zurich, Switzerland) focused her presentation on gene regulation in the female gametophyte and during early seed development by analyzing interaction partners and direct targets of the PRC2 methyltransferase MEDEA (MEA). Previous works have implicated MEA in the repression of seed development before fertilization and in endosperm cellularization (Chaudhury et al., 1997; Grossniklaus et al., 1998; Köhler et al., 2003a). The new unpublished data indicate that MEA interacts with histone deacetylases (HDACs), and that plants depleted in *HDACs* display similar abnormal phenotypes as *mea* mutants, suggesting an interplay between histone methylation and acetylation during early seed development.

The double fertilization process of plants generates an additional complication in the understanding of trans-generational inheritance and maternal and paternal contributions to the next generation. Thus, being able to distinguish events taking place in the endosperm from other plant tissues will be crucial to understand the peculiarities of this triploid tissue. An exciting technical advance in this direction was presented by Vikash Kumar Yadav (Uppsala, Sweden). He performed modified high-throughput chromatin conformation (mHi-C) on purified endosperm nuclei isolated by the INTACT method (Moreno-Romero et al., 2017), thus enabling Hi-C analysis on a limited number of nuclei. With this technique, he was able to observe elevated chromatin interaction levels in endosperm tissue compared to leaf tissue and discover that self-looping genes are on average expressed at a higher level compared to non-self-looping genes.

Heinrich Bente (Vienna, Austria) focused his presentation on yet another aspect of epigenetic inheritance, the phenomenon of paramutation, characterized by interallelic communication between epialleles at a single locus that results in stable and heritable silencing. Employing an epigenetically regulated resistance marker for hygromycin in Arabidopsis, Heinrich and his co-workers found that paramutation becomes apparent in F2 progeny of tetraploid hybrids but not in diploid ones. Small RNA profiles differ between the two epialleles, as do DNA methylation and chromatin marks. The fact that the paramutation is not observed at low temperatures, where also small RNA production is reduced (Baev et al., 2014), supports the assumption that small RNAs may be involved in paramutations.

Before epigenetic marks such as DNA methylation can be inherited between generations, they need to be maintained during cell divisions in the parents. Especially for asymmetric CHH methylation, maintenance is coupled to RdDM (Law and Jacobsen, 2010). Gergely Molnar (Tulin, Austria) reported the characterization of *freak show* (*fks*), a novel missense mutant of the RNA Polymerase V-specific subunit NRPE5A (Ream et al., 2009). The mutation displayed loss of transposon silencing due to generally reduced CG DNA methylation as well as hypermethylation at other loci, together leading to abnormal phenotypes, including flowering time defects and homeotic transformations. The findings seem to contrast canonical RNA Pol-V function in RdDM only, which mainly affects CHG and CHH methylation, and suggest a connection between an RdDM component and CG methylation maintenance.

## Session 3: Making Variations of Chromatin—Incorporating Bricks of Different Colors

To modulate nucleosome properties, including DNA accessibility and interactions between nucleosomes or even chromatin fibers, different histone variants can be incorporated. Recent years have seen accumulating evidence for the functional importance of these different histone variants for processes ranging from gene expression control and reprogramming to DNA repair processes in mammals and plants (Jiang and Berger, 2016; Buschbeck and Hake, 2017; Dabin and Polo, 2017).

Intriguing examples for these roles, reported by Anna Schmücke (Vienna, Austria), are the plant-specific histone variants H2A.W.6, H2A.W.7, and H2A.W.12, highly enriched in heterochromatin and involved in chromatin fiber–fiber interactions (Yelagandula et al., 2014). These histone variants are distinguished by a highly conserved KSPKK motif in their C-terminal tail. In response to DNA damage in heterochromatin, one of the three H2A.W variants, namely H2A.W.7, is phosphorylated at its SQE motif, and this phosphorylation is required for an appropriate DNA damage response (DDR) (Lorković et al., 2017). New evidence now indicates that only H2A.W.6, and not H2A.W.7, is phosphorylated in the conserved KSPKK motif in a cell cycle-dependent manner in Arabidopsis. Through a synthetic approach in fission yeast, she demonstrated that the phosphorylation of the KSPKK motif in addition to the phosphorylated SQE motif impairs a proper DNA damage response. This exemplifies a highly complex relationship between histone variants, their post-translational modification status, and their biological function. Another interesting H2A variant is H2A.Z, which has been associated both with transcriptional activation and repression depending on its position within a gene (Coleman-Derr and Zilberman, 2012; Sura et al., 2017). Wiam Merini (Seville, Spain) presented recent data resolving part of the mystery of this dual role of H2A.Z in transcription. She showed that, similar to canonical H2A, H2A.Z can be mono-ubiquitinated by PRC1 and that this post-translational modification plays an important role in transcriptional repression independent of PRC2 activity (Gómez-Zambrano et al., 2019). Indeed, complementation with a ubiquitination-resistant H2A.Z protein failed to rescue expression of upregulated genes in *h2a.z* mutant plants revealing the importance of H2A.Z ubiquitination. In contrast, H2A.Z ubiquitination seems to be dispensable to the rescue expression of the genes downregulated in *h2a.z* mutant plants; these genes may simply require H2A.Z incorporation. Alternatively, other post-translational modifications may play a role; in yeast, H2A.Z is acetylated by the NuA4 complex (Lu et al., 2009). Indeed, the confirmation that H2A.Z acetylation occurs in plants was provided by José A. Jarillo (Madrid, Spain). He studied the plant homologues of YEAST ALL1-FUSED GENE FROM CHROMOSOME 9 (YAF9) proteins, which are common components of the SWR1 complex involved in H2A.Z deposition and the NuA4 complex. In the absence of YAF9 proteins, H2A.Z acetylation is reduced at the *FLC* chromatin, and *FLC* expression is repressed, while H2A.Z incorporation as such is unaffected at this locus (Crevillén et al., 2019).

Given the emerging roles of the different histone variants in gene expression control and DNA repair reported at this conference, it becomes clear that histone deposition needs to be tightly controlled in time and space, and histone chaperones play an important role in this process. As an example, loss of H3 histone chaperones, such as HISTONE REGULATOR A (HIRA) (Nie et al., 2014; Duc et al., 2015) and the Arabidopsis ALPHA THALASSEMIA-MENTAL REDARDATION X-LINKED (ATRX) homologue (Duc et al., 2017), which function in complementary pathways of histone H3.3 deposition, results in altered gene expression. Aline V. Probst (GReD, France) discussed work from her laboratory, showing that ATRX loss-of-function affects H3.3 deposition at genes characterized both by elevated H3.3 occupancy and high expression levels, whereas *hira* mutants show reduced nucleosomal occupancy both at genes and in heterochromatin translating into reactivation of transposable elements. While some H3 histone chaperones are highly conserved, species-specific chaperones deposit the centromeric histone CenH3 (Müller and Almouzni, 2014). So far, the factor responsible for escort and deposition of plant CenH3 has remained enigmatic. Inna Lermontova (Gatersleben, Germany) reported on the collaborative effort to search for histone CenH3 interactors and the identification of the plant homologue of NUCLEAR AUTOANTIGENIC SPERM PROTEIN (NASP) as a CenH3 binding protein. Previously shown to bind histone H3 monomers or H3-H4 dimers (Maksimov et al., 2016), the nuclear NASP protein interacts both with the N-terminal tail as well as with the histone fold domain of CenH3 and reduced *NASP* expression negatively affects CenH3 levels, suggesting that NASP functions as a CenH3 escort protein (Le Goff et al., 2019).

## Session 4: a Tribute to Lars Hennig

Professor Lars Hennig passed away last year, leaving a gap in the fields of chromatin biology and plant development (Mozgová et al., 2018a). Session 4 of the meeting gave a homage remembering him, not only as a valuable colleague, friend, and mentor, but also by highlighting his scientific contributions and how his work will impact future research.

**Figure 2:**
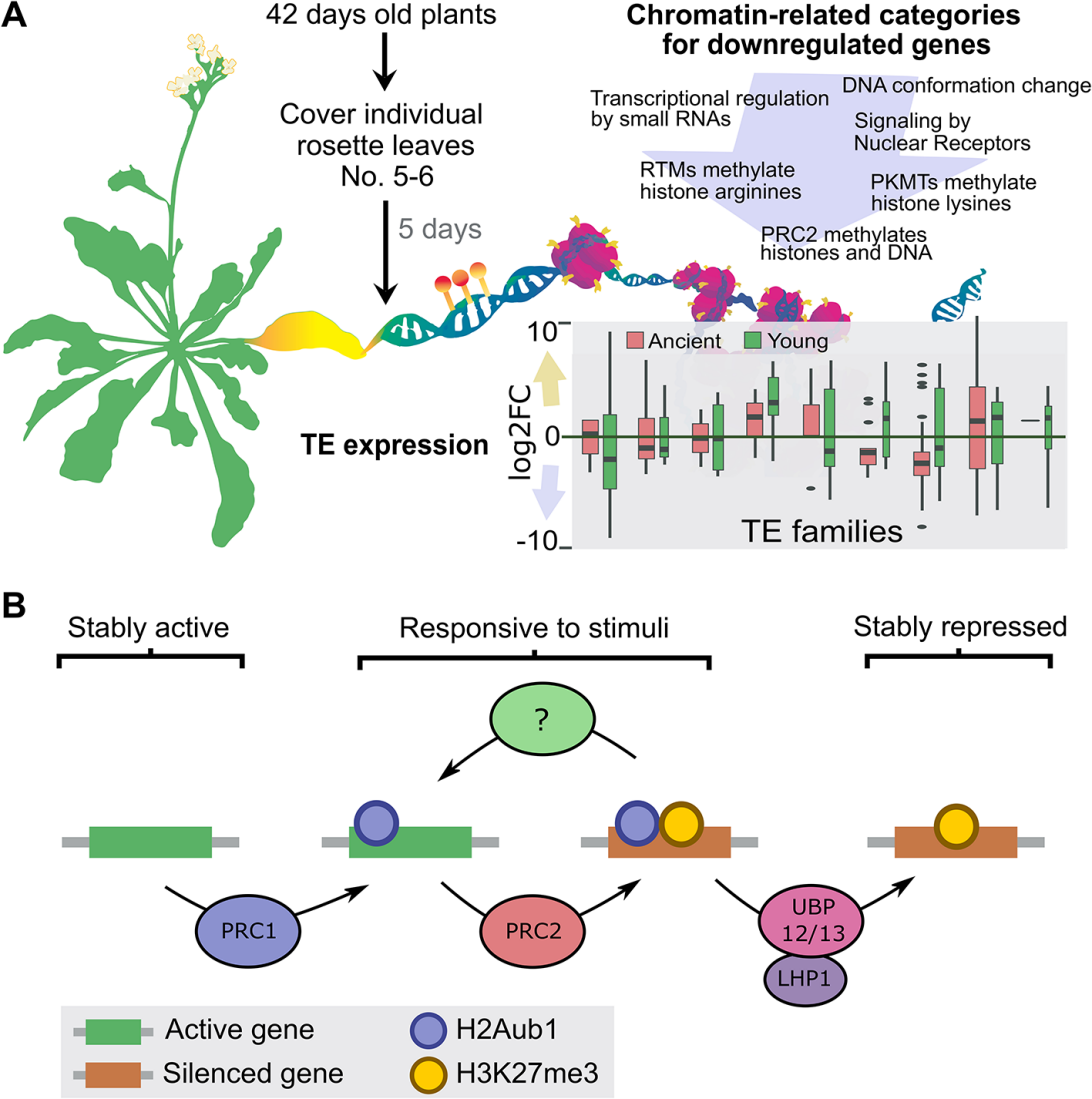
Overview of recent contributions from former Lars’ PhD students. **(A)** Dark-induced senescence causes localized changes in DNA methylation in Arabidopsis. Senescence was induced by covering individual Arabidopsis leaves. The yellowing of the covered-senescent leaves was accompanied by changes in the expression of transposable elements that depending on the TE family can be unaltered, up- or downregulated. Moreover, GO and pathway categories related with the maintenance of chromatin structure were enriched among the downregulated genes (for the complete analysis see Trejo-Arellano et al., 2019). Overall, the global DNA methylation landscape of the senescent leaves remained remarkably stable with only few localized DNA methylation changes detected, particularly in the CHH context **(B)** Working model for the UBP12/13-mediated gene repression. PRC2 causes silencing *via* deposition of H3K27me3, which in the majority of the cases is dependent on PRC1. However, by a mechanisms that remain to be resolved the product of PRC1 activity, H2Aub1, also creates an unstable state in which genes can be rapidly reactivated in response to a stimulus. Stable repression requires removal of H2Aub1 by LHP1-interacting UBP12/13. Figure courtesy of **(A)** M. Trejo, designed of Paulina Velasco, and **(B)** L. Kralemann.

Among many other topics, one of Lars’ main interests were histone variants and chaperones. He contributed to the identification of MULTICOPY SUPRESSOR OF IRA 1 (MSI1) as one subunit of the CHROMATIN ASSEMBLY FACTOR 1 (CAF-1) chaperone complex (Hennig et al., 2003). Lars further showed that transgenerational aggravation of the CAF-1 mutant phenotype was related to a global change in DNA methylation (Mozgová et al., 2018b). Following this curiosity on DNA methylation levels during development, Minerva Trejo-Arellano (Uppsala, Sweden), a former PhD student of Lars, reported on changes of DNA methylation during dark-induced leaf senescence (**Figure 2A**). She showed that senescent leaves had expanded chromocenters, which is indicative of heterochromatin de-condensation. These chromatin changes were accompanied by a concerted downregulation of genes involved in epigenetically mediated silencing pathways and a deregulation of transposable elements. Surprisingly, no genome-wide changes in DNA methylation were detected, only localized differentially methylated regions (DMRs), especially in the CHH context (Trejo-Arellano et al., 2019). Among the epigenetic changes that occur during developmental transitions, Lars soon focused his attention on Polycomb activity. He contributed to the identification of MSI1 as part of the PRC2 (Köhler et al., 2003b) and explored its role in embryo-to-seedling transitions, a work developed by Iva Mozgová (České Budějovice, Czech Republic) during her postdoc in Lars’ group. She found that the characteristic embryonic phenotype of the double mutant of *clf* and *swinger* (*clf swn*) (Chanvivattana et al., 2004; Mozgová et al., 2017), which is affected in two of the three possible methyltransferases of PRC2, depends on the presence of sucrose. This finding fits with the idea that, during this developmental transition, plant nutrition shifts from heterotrophic to autotrophic growth. Following this research line, Iva presented a progressive degradation of chloroplasts and an increase in Reactive Oxygen Species (ROS) in *clf swn* and, accordingly, the mitigation of the phenotype under reduced light intensities. Therefore, these data suggest an unexplored role of PRC2 in mediating the establishment and/or maintenance of photoautotrophic growth in Arabidopsis.

To further understand the multiple functions of PcG proteins, Lars’ laboratory found a direct interaction between MSI1 and LIKE HETEROCHROMATIN PROTEIN1 (LHP1) (Derkacheva et al., 2013). Studying LHP1 protein interactors, UBIQUITIN SPECIFIC PROTEASES (UBP) 12 and 13 were found, and it was demonstrated that UBP12 mediates the deubiquitination of H2A (Derkacheva et al., 2016). However, it has been shown that H2A ubiquitination (H2Aub) by PRC1 is largely independent of PRC2 activity (Zhou et al., 2017). To further understand the link between H2Aub and H3K27me3, Lars’ former PhD student Lejon Kralemann (Uppsala, Sweden) presented a genome-wide analysis of these marks in mutants deficient for UBP12 and 13. The data suggest that H2Aub removal is required for preventing the loss of H3K27me3. In that model, LHP1 recruits UBP12/13 to deubiquitinate H2Aub and to stabilize H3K27me3-mediated repression (**Figure 2B**). Miyuki Nakamura (Uppsala, Sweden), a postdoc in Lars’ former group, reported another role of LHP1 through its interaction with DEK proteins (Derkacheva et al., 2013), which are linked to chromatin and associated with DNA topoisomerase 1α (TOP1α) (Waidmann et al., 2014). Miyuki presented that DEKs genetically interact with LHP1 by enhancing the early flowering of the *lhp1* mutant, which is similar to what occurs in the *top1α lhp1* mutant (Liu et al., 2014). She proposed that LHP1 interaction with DEKs and TOP1α is important for PcG target gene regulation. These works exemplify the direction where Lars’ research has lead the PcG field: finding new players of PcG activity and identifying mechanisms for target-specific PcG recruitment. In that direction, Justin Goodrich (Edinburgh, United Kingdom), through a second invited lecture, presented new data about *ANTAGONIST OF LHP1* (*ALP1*), which was identified in a suppressor screening of the *clf* mutant (Liang et al., 2015). The interaction of ALP1 with PRC2 depends on ALP2, which interacts directly both with ALP1 and with MSI1, a core subunit of PRC2. To explain PcG antagonist function, Justin proposed that ALP1/ALP2 could compete for the core PRC2 complexes with other PcG “accessory proteins”. Interestingly, ALP proteins are likely inactive Harbinger-type transposases that are already demonstrated for ALP1 (Liang et al., 2015). As Harbinger transposases are encoded as part of the sequence of the ‘cut-and-paste’ *Harbinger* transposon superfamily (Kapitonov and Jurka, 2004), this is an example of how transposon domestication could provide novel genes for the hosts, in particular as components of PRC2.

## Session 5: an Open View on Chromatin

Session 5 focused on different mechanisms that are involved in inducing a more relaxed and open chromatin structure, which usually correlates with an active transcription. For instance, Julia Engelhorn (Cologne, Germany) presented a very elegant approach in which Fluorescence Activated Cell Sorting (FACS) was combined with the Assay for Transposase-Accessible Chromatin with high-throughput sequencing (ATAC-seq), which allowed for the creation of maps with higher resolution than with DNase-seq from low cell numbers. Lines expressing the *pDORNRÖSCHEN-LIKE::GFP* in the *apetala1 cauliflower* mutant background were used (Wellmer et al., 2006), which allows for cell sorting of identical and highly synchronized Lateral Organ Founder Cells (LOFCs) (Frerichs et al., 2016). LOFCs-associated changes in chromatin accessibility were positively associated with transcriptional changes. In addition, highly accessible chromatin to the transposase corresponded well with previously described enhancer and conserved transcription factor (TF)-binding elements in promoters. These results also demonstrated that this approach can be further applied for genome-wide identification of novel transcriptional enhancers in plant specific cells (Frerichs et al., 2019).

Genome-wide approaches were also used to identify light-induced chromatin dynamics that occur at a very specific developmental switch, such as photomorphogenesis, which corresponds to the first perception of light after germination (Casal, 2013; Wu, 2014; Seluzicki et al., 2017). DE-ETIOLATED1 (DET1) is an atypical and conserved DAMAGED DNA BINDING PROTEIN 1 (DDB1)-CULLIN4 Associated Factor (DCAF) involved in the transcriptional reprogramming that occurs during photomorphogenesis (Chory et al., 1989; Pepper et al., 1994; Schroeder et al., 2002; Ma et al., 2003). Sandra Fonseca (Madrid, Spain) showed that DET1 and light control genome-wide levels and distribution of H2B ubiquitination (H2Bub) indirectly through degradation of a deubiquitination trimeric module (DUBm). One of the components of DUBm is UBP22, which acts as a major H2B deubiquitinase in the plant. Thus, DET1-mediated proteolytic degradation of DUBm is essential for chromatin reprogramming during photomorphogenesis (Nassrallah et al., 2018).

High Mobility Group A (HMGA) proteins have also been proposed to create a more permissive chromatin structure competing with linker histone H1 (Catez and Hock, 2010; Ozturk et al., 2014). Simon Amiard’s (Clermont Ferrand, France) presentation focused on GH1-HMGA1 and GH1-HMGA2 proteins, which comprise a conserved central globular domain (GH1) as well as AT-hook domains (Kotliński et al., 2017). Both GH1-HMGA1 and GH1-HMGA2-GFP fusion proteins are present in interphase and mitotic nuclei but are excluded from chromocenters and centromeres, and protein–protein interaction studies indicate possible GH1-HMGA1 homodimerization and heterodimerization with GH1-HMGA2. Mutants affected in the *GH1-HMGA1* gene were impaired in development, with an overall size reduction due to smaller roots and leaves and a decrease in stem length, while *gh1-hmga2* mutants were phenotypically normal. *gh1-hmga1* mutants also showed shorter telomeres as a result of telomere instability (Charbonnel et al., 2018), and a transcriptome analysis of *gh1-hmga1* mutants suggested a contribution of GH1-HMGA1 proteins to gene expression control.

Another epigenetic hallmark of active chromatin is H3K4me3. In yeast, SET DOMAIN GROUP 1 (SET1) adds this mark as part of COMPlex of proteins ASsociated with SET1 (COMPASS). Another subunit of this complex, Swd2, is needed for the recruitment of COMPASS to specific chromatin domains enriched in H2Bub (Sun and Allis, 2002; Kim et al., 2009). In Arabidopsis, a more complex scenario may exist since H3K4me3 can be placed by different histone methyltransferases (Baumbusch et al., 2001; Thorstensen et al., 2011; Zhang and Ma, 2012), and the function of At-COMPASS-like complexes have not been fully characterized yet (Fromm and Avramova, 2014; Xiao et al., 2016). Clara Bourbosse (Paris, France) reported recent results that showed that SET DOMAIN GROUP 2 (SDG2)/ARABIDOPSIS TRITHORAX 3 (ATX3), which has a main role in the deposition of H3K4me3 in Arabidopsis (Berr et al., 2010; Guo et al., 2010), binds to SWD2-like b (S2Lb), which interacts with core subunits of AtCOMPASS in a high-molecular weight complex. In addition, S2Lb, together with SDG2, is required for deposition of H3K4me3 and directly targets highly expressed genes. However, mutations in *S2Lb* affect the steady state levels of only a few of its target genes. Therefore, as part of AtCOMPASS, S2Lb may be required for appropriate transcriptional dynamics but is not essential for gene expression. Interestingly, S2Lb recruitment and H3K4me3 deposition at target genes are independent of H2Bub, indicating that AtCOMPASS-S2Lb activity does not require H2Bub in contrast to yeast. Whether there is a crosstalk between this histone mark and other methyltransferases is still an open question (Fiorucci et al., 2019).

## Session 6: Friends and Foes —Chromatin Interactors and Transcription Regulation

Whether it is by changing large-scale chromatin conformation, nucleosome composition, and occupancy or histone post-translational modifications, chromatin regulation can impact plant development in a variety of ways, as highlighted in the previous sessions. This complexity becomes more evident when, despite being highly conserved among the plant species, the function of chromatin regulators, as well as their target genes, also depends on the context in which they act (Hennig et al., 2005; Merini et al., 2017). The last session of the meeting focused on the interplay between different chromatin regulators and accessory factors as well as their role in transcription regulation and impact on plant development.

Chromatin-based regulation allows us to quickly and reversibly switch genes on and off through the concerted action of antagonistic regulators. One example was presented by Cristel Carles (Grenoble, France) with her latest work on ULTRAPETALA1 (ULT1). It was known that ULT1 antagonizes the activity of PRC2 and regulates levels of H3K27me3 at genes involved in flowering and meristem determination (Carles and Fletcher, 2009). The new work showed that ULT1 genome-wide targets strongly overlap with those of the H3K27me3 methyltransferase CLF but not with the genes targeted by the demethylase RELATIVE OF EARLY FLOWERING 6 (REF6). ULT1 interacts with RNA Pol II (RNAPII) and several chromatin remodelers, suggesting that it might be involved in their recruitment, preventing binding of PcG proteins at specific loci.

TFs have also been shown to play a role in recruiting chromatin-associated regulators to control different aspects of plant development (Vachon et al., 2018). Pawel Mikulski (Norwich, United Kingdom) presented his work on VP1/ABI3-LIKE 1 (VAL1), a transcriptional repressor that promotes histone deacetylation at the *FLC* locus and is required for PRC2 nucleation in cold-induced vernalization (Questa et al., 2016). VAL1 was found to interact with subunits of the PRC1 (Yang et al., 2013; Questa et al., 2016), PRC2 (Chen et al., 2018), and LHP1 (Yuan et al., 2016), but no differences were observed in H2Aub in the mutants. Interestingly, the authors found that VAL1 influences nucleosome mobility around the region of the PRC2 nucleation site, suggesting it may act through the recruitment of chromatin remodelers. Other ways to achieve specificity include the formation of alternative chromatin-associated complexes or the interaction of core components with specific accessory proteins (Förderer et al., 2016). One example of the former was presented by Hernan Lopez-Marin (Cologne, Germany) with the identification of SUPER DETERMINANT 1 (SDE1), a new regulator of axillary meristem initiation in tomato. The *sde1* mutation was mapped to a gene closely related to the PRC1 core components BMI1 and RING1 but which lacks the RING-finger domain required for depositing H2Aub (Buchwald et al., 2006). SDE1 interacts with LHP1, another component of the PRC1, suggesting it may be part of a new PcG complex involved in regulating axillary meristem initiation in tomato. Additionally, Sara Farrona (NUI, Galway) presented work from her laboratory on the identification of UBP5 as a new interactor of PWO1 and PRC2 subunits. As discussed in the first session of the meeting, PWO1 is itself an interactor of PRC2 methyltransferases and is involved in recruiting CLF to foci associated with the nuclear lamina (Hohenstatt et al., 2018; Mikulski et al., 2019). *ubp5* mutants show a pleiotropic phenotype and de-repression of several meristem identity genes, known targets of PRC2, suggesting that UBP5 acts together with PcG proteins to regulate plant development.

Chromatin environments are also crucial for correct gene expression since they can modulate RNA polymerase II (RNAPII) and TF accessibility to target DNA. An interesting example was presented by Sebastian Marquardt (Copenhagen, Denmark), who showed that the histone chaperone complex FACT is required for the repression of cryptic intragenic Transcriptional Start Sites (TSSs) during RNAPII-mediated transcription. In their repressed state, these TSSs are enriched in H3K4me1, a hallmark for RNAPII elongation, while, in the *fact* mutants, they show increased levels of H3K4me3, similar to promoter TSSs, indicating a role for FACT in the regulation of transcript isoform diversity (Nielsen et al., 2019). Moreover, a computational approach presented by Dmitry Lapin (Cologne, Germany) helped to define chromatin features predicting dependency of gene expression on the immunity regulator Enhanced Disease Susceptibility 1 (EDS1) in Arabidopsis. Machine-learning methods were used to test whether this dependency can be inferred from binding of TFs and occupancy of histone modifications from public ChIP-seq data. A neural network model provided the highest accuracy (up to 85%). Under non-stress conditions, EDS1-dependent loci have low H3K36me3 and RNAPII levels. Authors proposed that initial chromatin status contributes to the specificity of gene expression regulation in immunity. On the other hand, taking advantage of epigenetic hybrids (epiHybrids) from crosses with *decrease in dna methylation1* (*ddm1*)-derived epigenetic recombinant inbred lines (epiRILs), Ioanna Kakoulidou (Munich, Germany) showed that chromatin states can also impact subsequent generations. Previous work from the laboratory had shown that epiHybrids exhibit strong heterosis in several developmental traits, which correlates to DMRs in the parental lines (Lauss et al., 2018). Recently, the authors have used a high throughput phenotyping system to analyze 382 epiHybrids, and they were able to confirm that epigenetic divergence in the parents is sufficient to cause heterosis in the progeny. Future methylome, transcriptome, and small RNA-seq analyses of these epiHybrids are expected to contribute to a better understanding of how the parental epigenetic states affect the progeny.

## Conclusions and Perspectives

In summary, the EWPC2019 encouraged discussion about the most recent advances in epigenetics, chromatin-related mechanisms, and nuclear architecture in relation to the regulation of transcription and its impact on plants traits. Particularly, how the nuclear space is organized and how specific histones and structures within the nucleus, such as the nucleolus and the nuclear periphery, relate to specific chromatin domains was thoroughly discussed in various talks. However, we are still far from understanding the complexity that is shrouded by the nuclear envelope. The extent of interplay between DNA methylation, various histone modifications, histone variants, and regulatory RNAs taking place during epigenetic inheritance processes remains to be elucidated. Likewise, much remains to be understood on the importance of core histone variants and their chaperones in chromatin structure through the control of nucleosome assembly and occupancy or the role of linker histones and other dynamic DNA-binding proteins. While histone modifications have so far rarely been considered in a variant-specific manner, combinations of histone variants with their particular marks constitute an additional layer of complexity to fine-tune chromatin regulation that is now just emerging and that will most certainly require further studies. While different presentations exposed the complexity of chromatin-based regulatory mechanisms in plants, it became clear that we need to investigate how chromatin-associated proteins are regulated in different tissues, developmental stages, and under specific environmental conditions, in order to fully understand their role in transcriptional regulation. Novel technical advances making use of CRISPR/Cas9 based strategies or new developments in 3C techniques together with a deeper characterization of multi-subunit complexes and their functions will help to better our understanding of the organization of plant genomes and nuclear protein networks in the near future. Simultaneously, studying the interplay between different regulators with the help of emerging technologies, such as the development of imaging and image-processing solutions that take into account the challenges of plant systems (Dumur et al., 2019) and other cell-specific techniques, will certainly yield important new findings. Finally, while most work presented at the meeting used Arabidopsis as a model system, the fundamental mechanisms identified might in the future be applied to crop species by, for example, exploiting natural epigenetic diversity in plant breeding or induced epigenetic variation involved in stress priming and memory (Springer and Schmitz, 2017; Mozgová et al, 2019; Forestan et al., 2019). We expect to see some of these questions addressed in the future and exciting new data on chromatin regulation in model and crop plants to be presented in forthcoming EWPCs.

The memory of Lars Hennig imbued specifically one of the meeting sessions but was also present in many other talks, demonstrating that the contributions of this excellent scientist and mentor will last over time. His work had a tremendous impact on the understanding of chromatin regulation and plant development, particularly concerning our knowledge of the PcG pathway, and will perpetuate through the ongoing contributions of many of his alumni who are still actively investigating these questions.

## Author Contributions

SF and AP created **Figure 1**. JM-R edited **Figure 2**. Each author (JM-R, K, JE, IT, AP, and SF) wrote one session. JM-R, AP, and SF wrote introduction and conclusions. All authors contributed to edit text and figures. IT edited references. SF coordinated the manuscript.

## Funding

JM-R is supported by funding from the European Union’s Horizon 2020 research and innovation program under the Marie Skłodowska-Curie grant agreement No 797473, financial support from the Spanish Ministry of Economy and Competitiveness through the “Severo Ochoa Program for Centres of Excellence in R&D” 2016-2019 (SEV‐2015‐0533), and by the CERCA Programme/Generalitat de Catalunya. AP, K, and SF acknowledge networking support from the COST Action CA16212 “Impact of nuclear domains on gene expression and plant traits (INDEPTH).” The EWPC was supported by grants from The German Research Foundation (TU126/14-1) and The Company of Biologists (EA1772). The organizers also acknowledge the generous contributions from industrial sponsors Zeiss Microscopy GmbH (Germany), Diagenode SA (Belgium), and Dispendix GmbH (Germany).

## Conflict of Interest

The authors declare that the research was conducted in the absence of any commercial or financial relationships that could be construed as a potential conflict of interest.
